# TLR-independent activation of NK cells during systemic inflammation

**DOI:** 10.1186/cc14004

**Published:** 2014-12-03

**Authors:** O Rasid, J-M Cavaillon

**Affiliations:** 1 Cytokines and Inflammation, Institut Pasteur, Paris, France

## Introduction

During the course of systemic inflammation, most of the immune cell types get activated to a certain degree as part of, or contributing to, the cascade of physiopathological events. Whether for some cells, classically phagocytes of the innate immune system, it is clear that direct sensing of pathogen-associated molecular patterns leads to activation initiating systemic inflammation, the picture is not so clear for natural killer (NK) cells. While NK cells have been shown to express toll-like receptors (TLR), the role of these receptors on NKs during systemic inflammation has not been directly addressed.

## Methods

To directly assess the role of TLR expression on NK cells we used an adoptive transfer model in which NKs purified from the spleens of WT, TLR4KO and TLR2/4DKO mice were transferred intravenously to RAG2^-/-^γc^-/- ^(devoid of T, B and NK cells). Five days after reconstitution the mice were challenged intraperitoneally with conventional or TLR-grade lipopolysaccharide (LPS). Immune cell activation and production of IFNγ by NK cells was determined after 6 hours by FACS analysis.

## Results

We observed no differences in reconstitution of the recipient mice with NK cells from different backgrounds suggesting no difference in trafficking and survival of the transferred cells. At 6 hours after LPS challenge, WT, TLR4KO or TLR2/4DKO NK cells recovered from the spleen and lungs of RAG2^-/-^γc^-/- ^mice showed comparable levels of CD69 activation marker expression. Intracellular labeling for IFNγ in NK cells also revealed no significant differences.

## Conclusion

Whether there is a role for direct TLR signaling on NK cells remains the objective of further investigations; however, our data show that in the course of a systemic inflammatory process, like endotoxinemia, the expression of TLR2 and TLR4 by NK cells makes no difference in terms of their activation and secretion of IFNγ.

